# The efficacy and safety of selective RET inhibitors in RET fusion-positive non-small cell lung cancer: a meta-analysis

**DOI:** 10.1007/s10637-023-01390-3

**Published:** 2023-08-21

**Authors:** Jun-yi Ke, Shu Huang, Zhi-tao Jing, Min-chao Duan

**Affiliations:** 1 Guangxi Medical University, Nanning, People’s Republic of China; 2 Department of Respiratory Medicine, The Second Affiliated Hospital of Guangxi Medical University, Nanning, People’s Republic of China; 3 Department of Respiratory Medicine, Wuming Hospital of Guangxi Medical University, Nanning, People’s Republic of China

**Keywords:** Meta analysis, Selective RET inhibitors, RET fusion-positive, Non-small cell lung cancer, Pralsetinib, Selpercatinib

## Abstract

Rearranged during transfection (RET) fusion-positive occurs in approximately 2% of non-small cell lung cancer (NSCLC). This mutation often predicts metastasis risk and poor prognosis, and current mainstream therapies provide limited patient benefit. Selective RET inhibitors Pralsetinib and Selpercatinib are targeted drugs approved by the US Food and Drug Administration for treating RET-mutated tumors. The phase I/II clinical trial results of their treatment of NSCLC have been published. However, the clinical effect of selective RET inhibitors on RET fusion-positive NSCLC remains controversial. ***Purpose*** Meta-analysis was performed to investigate the efficacy and safety of selective RET inhibitors in treating RET fusion-positive NSCLC. ***Methods*** Qualified literature was searched in Pubmed, Cochrane Library, Embase, and Web of Science. Outcomes included objective response rate (ORR), median progression-free survival (mPFS), disease control rate (DCR), intracranial ORR, and adverse events. Stata 15.1 software was used to analyze the data. ***Results*** A total of 8 studies were included in this meta-analysis. The combined results showed that the ORR of patients treated with selective RET inhibitors was 67% (95% confidence interval:0.64 to 0.70, *P* < 0.01), DCR was 92% (95%*CI*: 0.91–0.94, *P* < 0.01), the mPFS was 16.09 months (95%CI: 11.66–20.52, *P* < 0.01). In treated patients with RET mutation, the intracranial ORR was 86% (95%*CI*:0.74 ~ 0.96, *P* < 0.01). ORR in untreated patients was more effective than untreated patients [HR = 0.44 (95%*CI*: 0.35–0.56, *P* < 0.01)]. The major adverse events (grade 3–4) are neutropenia (13%) and anaemia (13%). ***Conclusions*** Selective RET inhibitors Pralsetinib and Selpercatinib have shown a good effect on RET fusion-positive NSCLC, with a low incidence of adverse events.

**Supplementary Information:**

The online version contains supplementary material available at 10.1007/s10637-023-01390-3.

## Introduction

At present, lung cancer is the malignant tumor with the highest incidence in humans and first cancer that leads to human death. According to statistics, in 2020, lung cancer cases globally reached 2.2 million, and deaths reached 1.8 million, accounting for 18% of the total cancer deaths [1]. Although there are many pathological subtypes of lung cancer, small cell lung cancer and non-small cell lung cancer (NSCLC) are often used for pathological diagnosis to facilitate the selection of treatment for clinicians. Among them, NSCLC accounts for more than 80% of newly diagnosed lung cancer, and most patients are diagnosed with advanced lung cancer^1^. The treatment of advanced NSCLC mainly includes platinum-based combination chemotherapy and molecular targeted therapy, but the effect of the current treatments are not ideal [2]. Therefore, it is necessary to develop new and effective targeted therapies.

Rearranged during Transfection(RET) gene is a proto-oncogene that participates in various physiological processes of embryonic development by encoding one-way transmembrane receptor tyrosine kinase [3–5]. RET fusion refers to the juxtaposition of the 5’-terminal sequence of other genes with the 3’-terminal sequence of RET gene encoding tyrosine, which has been seen in a variety of solid tumors, among which NSCLC is the most common, and RET rearrangement can be detected in about 2% of NSCLC patients [6, 7]. The kinesin family 5B gene (KIF5B), Coiled-Coil Domain Containing six genes (CCDC6), and Nuclear receptor coactivator was also associated with 4 (NCOA4) and other partner gene fusion [8]. This mutation leads to non-ligand-dependent activation and abnormal expression of RET, forming a high level of active RET oncoprotein and multi-kinase signaling center, promoting the occurrence of NSCLC and being considered a target for the treatment of NSCLC [9]. In addition, RET fusion-positive NSCLC patients have an inadequate response to immunotherapy due to low tumor mutation load and low programmed cell death ligand 1 (PD-L1) expression [10]. Based on these characteristics, various molecular-targeted drugs have been applied to treat RET fusion-positive NSCLC.

Multiple kinase inhibitors (MKIs) were found to have significant effects on RET, vascular endothelial growth factor receptor-2(VEGFR2), The hepatocyte growth factor receptor (MET), The Multiple targets of tyrosine kinase, such as epidermal growth factor receptor (EGFR), have inhibitory effects [11], and are first used for molecular targeted therapy of advanced RET fusion-positive NSCLC. MKIs such as Cabozantinib, Vandetanib, and Lenvatinib have been tested in clinical trials to treat RET fusion-positive NSCLC. A phase II clinical trial conducted by Drilon [12] in the United States showed that Cabozantinib had an objective response rate(ORR) of only 28% in the treatment of RET-rearranged NSCLC. In the multi-center Phase II clinical trial conducted by Yoh^7^, among 17 patients, ORR in RET rearranged NSCLC treated by Vandetanib was 53%. In phase II multi-center clinical study of Lenvatinib in RET fusion-positive NSCLC reported by Hida [13], ORR was only 16% in 25 patients. At the same time, tertiary adverse events related to the drug occurred in 23 patients. The efficacy and safety of MKIs in the treatment of RET-mutated NSCLC were less than expected. Therefore, there is a need to find more effective and safer treatments to improve the prognosis of RET fusion-positive NSCLC patients.

Selective RET specific-tyrosine kinase inhibitors (RET-TKIs) Pralsetinib and Selpercatinib have been approved by the US Food and Drug Administration to treat RET fusion-positive NSCLC, showing highly selective tyrosine kinase inhibition. Selpercatinib is highly selective in inhibiting multiple RET mutations and has blood-brain barrier penetration, which plays a role in Brain metastases [14]. Pralsetinib selectively acts on various RET carcinogenic mutations and can be used in treating multi-kinase inhibitors and third-generation EGFR-TKIs-resistant lung cancer [15]. Multi-center, phase I/II clinical trials conducted by Drilon [16] and Gainor [17] have shown that Both Selpercatinib and Pralsetinib showed good tumor response in patients with RET rearrangement positive NSCLC and significantly extended disease-free survival. In order to more accurately and objectively evaluate the efficacy and safety of RET-TKIs in the treatment of RET fusion-positive NSCLC, our study combined the results of several studies to provide objective evidence of the efficacy and safety of RET-TKIs in the treatment of RET fusion-positive NSCLC.

## Methods

### Search strategy

The meta-analysis solution has been PROSPERO registered (CRD42022377465) on (https://www.crd.york.ac.uk/PROSPERO/). Use the following search terms: Non-small lung cancer, lung neoplasms, et fusion-positive, pralsetinib, non-small lung cancer, lung neoplasms, ret fusion-positive, pralsetinib, selpercatinib et al. searched in Pubmed, Embase, Cochrane Library and Web of Science databases in the form of subject terms and free words. The search period was from establishing the database to July 25, 2023. In addition, the references to be included in the study were also searched for supplementary literature.

### Selection criteria

Inclusion criteria: [1] The research objects are 18 years of age or older RET fusion-positive patients with non-small cell lung cancer [16, 2] Selective RET kinase inhibitors (Pralsetinib, Selpercatinib); [3] Outcomes included any of the following: mean progression-free survival (mPFS), ORR, disease control rate (DCR), Intracranial ORR, adverse events; [4] The study type was non-randomized controlled clinical trial; [5] ORR or Safety as the primary endpoint event;

Exclusion criteria: [1] duplicate literature; [2] non-English literature; [3] The research content is inconsistent; [4] Incomplete or unavailable research data; [5] Basic experiments, case cases, summaries, or abstracts of meetings;

### Data extraction

After literature retrieval, the required information was extracted according to the research design. The two researchers independently read the literature according to the inclusion and exclusion criteria and selected the corresponding information (J Y Ke, S Huang). If there are pending points, they will discuss them with the third researcher (Z T Jing) before deciding whether to include them in the study. The following information was extracted from the literature review: investigator name, publication period, study design, mPFS, ORR, DCR, Intracranial ORR, and adverse events.

### Quality evaluation

The Newcastle-Ottawa Scale (NOS) [18] was selected to evaluate the quality of the included studies. Studies with more than 4 stars were included in the meta-analysis (S1 Table).

### Statistical analysis

Stata 15.1 software was used to combine and meta-analyze the extracted data. Dichotomy variables were adopted at Hazard Ratio (HR) as the effective index, and continuous variables were adopted with Mean ± SD as the effective index. The data were extracted from the selected literature, and the *χ*^*2*^ test and *I*^*2*^ statistic were used to carry out the heterogeneity test. The heterogeneity was considered to be small when *P*>0.10 and *I*^*2*^≤50%. Meta-analysis results are reported with 95% confdence interval (CI). In this study, when *I*^*2*^≤50%, the random effects model was used for combined analysis, Otherwise, the fixed random effects model was used for meta-analysis. With bilateral *P* < 0.05 was considered statistically significant.

## Results

### Study selection

**Fig. 1:**
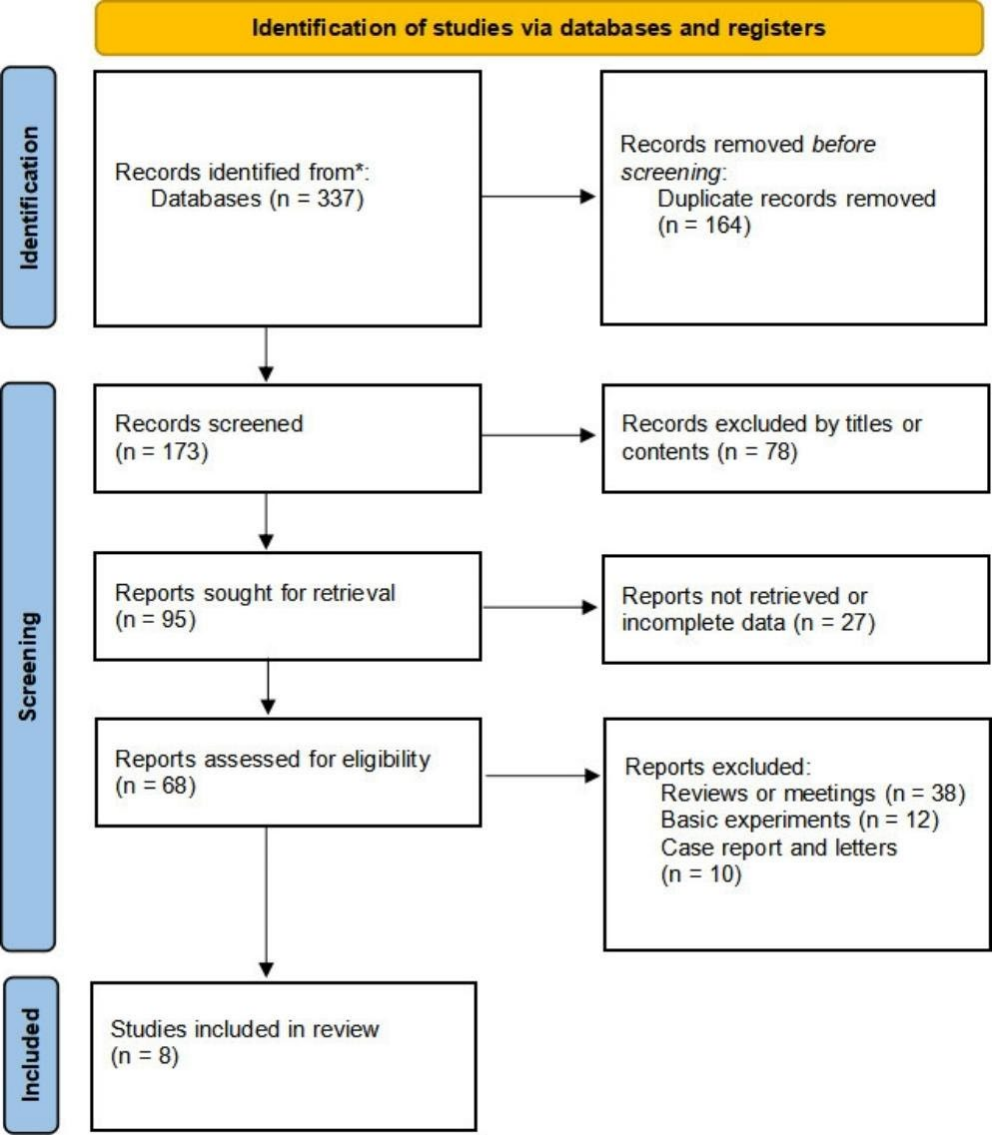
PRISMA flow diagram of the study selection process for the meta-analysis

After preliminary screening, two hundred ninety-three literature were included in the database (95 in Pubmed, 163 in Embase, 16 in Cochrane Library, and 63 in Web of Science), and 173 were included after removing duplicate literature. After screening strictly according to the inclusion and exclusion criteria, 8 qualified literature were included in the study. All included studies were Phase I/II clinical studies (Fig. 1; Table 1).

**Table 1:** Basic characteristics of the included literature in the meta-analysis

Study	Interventions	Patients, n (treated/untreated)	Age, years (range)	Female, n (%)	RET-fusion gene, n(%) (RET-KIF5B fusion)	Primary endpoint	Quality assessment
Lu, 2022	Selpercatinib	47 (36/11)	26–72	26 (55.3)	-	ORR	7
Drilon, 2020	Selpercatinib	144 (105/39)	23–86	84 (58.3)	85 (59)	ORR	8
Gainor, 2021	Pralsetinib	121 (92/29)	53–69	61 (50.4)	89 (73.6)	ORR,Safety	8
Griesinger, 2022	Pralsetinib	233 (158/75)	26–87	122 (52.4)	164 (70.4)	ORR,Safety	8
Drilon, 2022	Selpercatinib	316 (247/69)	23–92	183 (57.9)	201 (63.6)	ORR	8
Illini, 2021	Selpercatinib	50 (13/37)	38–89	30 (60)	33 (66)	ORR	7
Meng, 2022	Selpercatinib, Pralsetinib	49 (37/12)	26–77	23 (46.9)	13 (26.5)	ORR	5
Zhou, 2023	Pralsetinib	68 (37/31)	26–79	40(58.8)	44(64.7)	ORR	8

### Tumor response

**Fig. 2:**
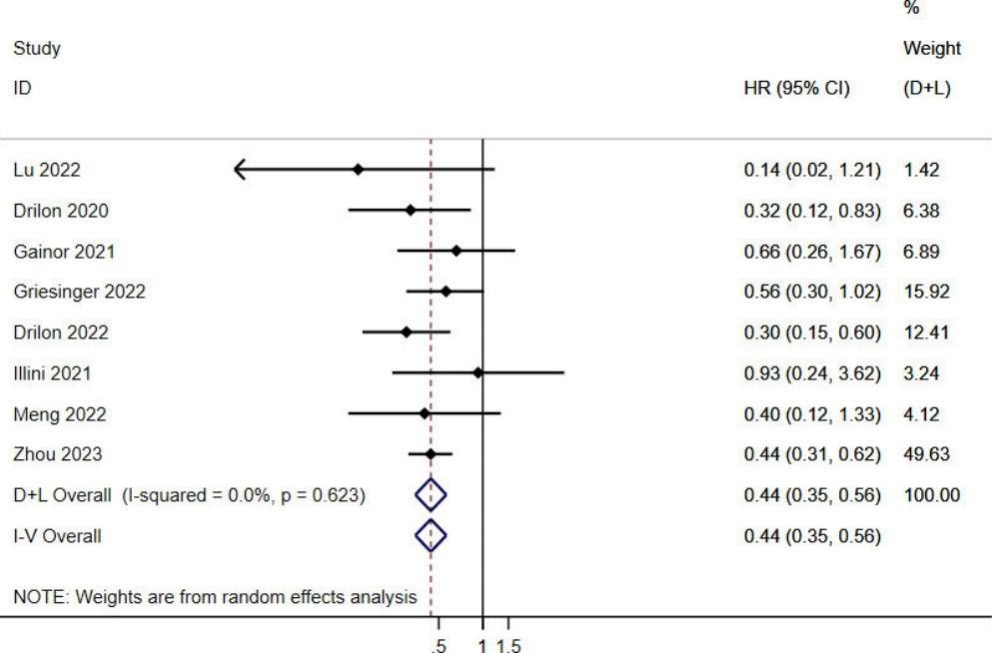
A double-limb meta-analysis of ORR after treatment with RET-TKIs in RET fusion-positive NSCLC patients treated or untreated

In this study, seven articles reported ORR in previously treated or untreated RET fusion gene-positive non-small cell lung cancer patients treated with RET-TKIs. After the heterogeneity test, *I*^*2*^ = 0.0%, and *P* = 0.623, indicating that the heterogeneity among the literature selected in this study was not statistically significant. The HR value of the combined ORR was 0.44 with a 95% confidence interval of 0.35 to 0.56 (*P* < 0.05). The results showed that the probability of achieving ORR in the previously treated patients was only 0.44 compared to the untreated patients, indicating that untreated RET fusion gene-positive NSCLC patients treated with RET-TKIs had better tumor response, ORR was better in RET fusion gene-positive NSCLC patients who had been untreated before (Fig. 2).

Further analysis of RET fusion gene-positive NSCLC patients who received treatment showed no heterogeneity among this literature (*I*^*2*^ < 50%, *P* > 0.1) and then merged ORR from this literature with a fixed effect model to obtain Effect Size (ES) directly. We found that ES of patients treated with PD-1/PD-L1, MKI, and chemotherapy was 57% (95%*CI*: 50-64%), 61% (95%*CI*: 53-69%), and 60% (95%*CI*: 53-67%), respectively. In untreated RET fusion-positive NSCLC patients, the ORR of RET-TKIs reached 79% (95%*CI*: 73-84%). It suggested significant differences in the ORR of RET-TKIs after receiving different pre-treatment regimens (Table 2, S1 Fig).

**Table 2:** meta-analysis of the effects of different earlier treatments on ORR of RET-TKIs

Treatment type	Numbers (N)	Patients (Events/All)	ES (95% *CI*)	Heterogeneity *I*^*2*^ (%)	*P* of ES
PD−1/PD-L1	5	119/209	57 (50 ~ 64)	0.00	*P* < 0.01^**^
MKI	5	100/165	61 (53 ~ 69)	0.00	*P* < 0.01^**^
Chemotherapy	3	111/186	60 (53 ~ 67)	0.00	*P* < 0.01^**^
Untreated	7	183/234	79 (73 ~ 84)	21.11	*P* < 0.01^**^

**P < 0.01

**Fig. 3:**
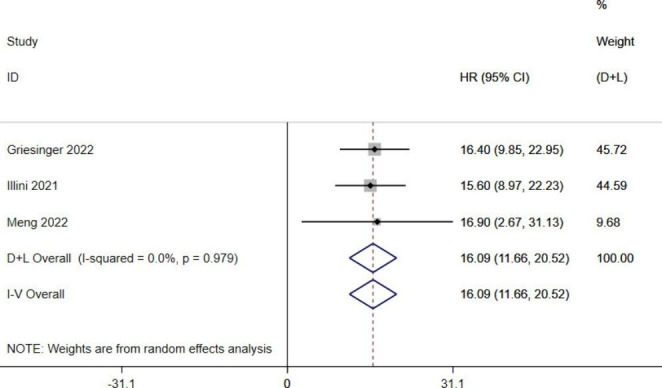
A single-limb meta-analysis of mPFS in RET-TKIs patients with RET fusion-gene positive NSCLC

This [19, 20, 21] meta-analysis showed that (*I*^*2*^ < 50%, *P* > 0.1), the mPFS of RET fusion-positive NSCLC patients treated with RET-TKIs were significantly prolonged to 16.09 months (95%*CI*: 11.66–20.52, *P* < 0.05) (Fig. 3).

**Fig. 4:**
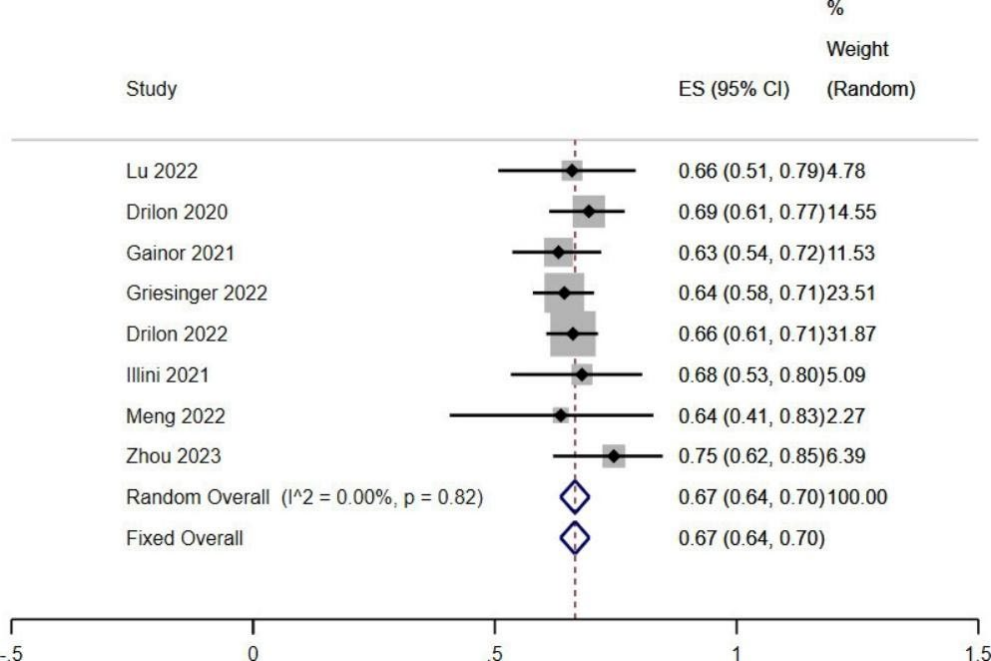
A single-limb meta-analysis of ORR in patients with RET fusion-gene positive NSCLC treated by RET-TKIs

This study included seven articles in the ORR of RET fusion-positive NSCLC patients treated with RET-TKIs. There was no significant heterogeneity among the included articles (*I*^*2*^ < 50%, *P* > 0.1). Fixed effects model analysis showed that the combined ORR was 0.67 (95%*CI*: 0.64–0.70, *P* < 0.05), suggesting that RET-TKIs have an excellent tumor response (Fig. 4).

**Fig. 5:**
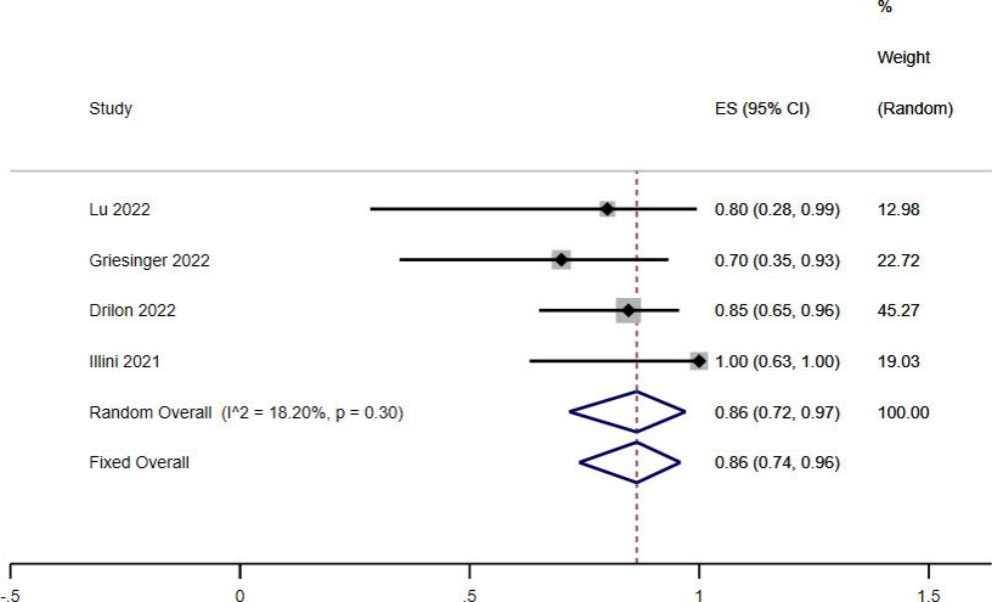
A single-limb meta-analysis of Intracranial ORR treated by RET-TKIs in patients with RET fusion-gene positive NSCLC

Intracranial ORR of RET fusion-positive NSCLC treated by RET-TKIs was included in 4 literature, and there was no heterogeneity among them (*I*^*2*^ < 50%, *P* > 0.1). Intracranial ORR after the fixed effects model was 0.86 (95%*CI*: 0.74 ~ 0.96, *P* < 0.05), indicating that RET fusion gene-positive NSCLC with intracranial metastasis still has an excellent response to drugs (Fig. 5).

**Fig. 6:**
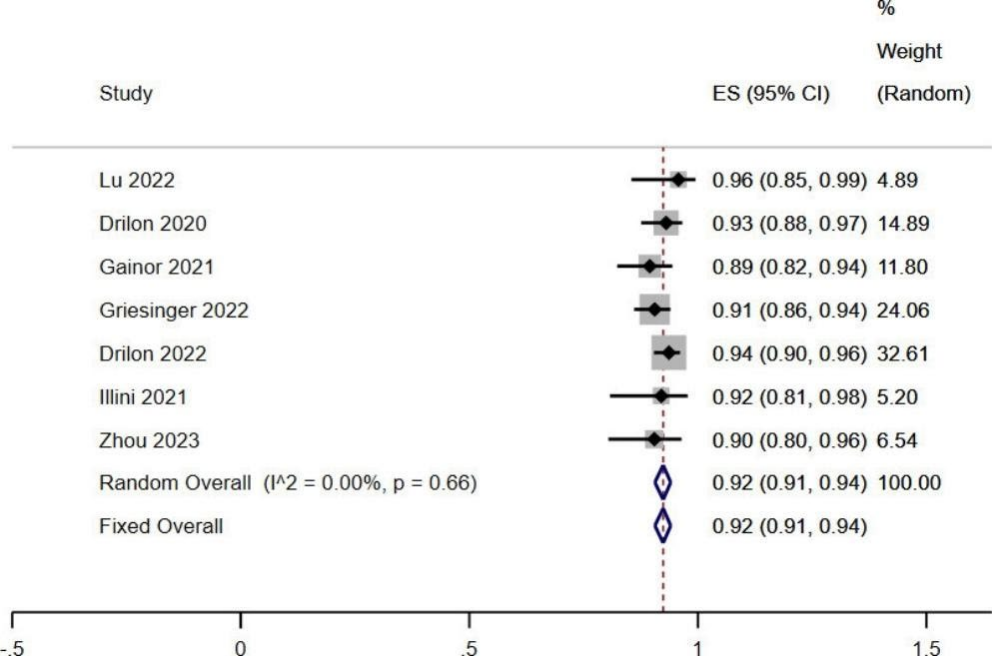
A single-limb meta-analysis of DCR in patients with RET fusion-gene positive NSCLC treated by RET-TKIs

A total of 7 studies were included in the DCR of RET fusion-positive NSCLC patients treated with RET-TKIs, and the heterogeneity of these 7 studies of literature was not statistically significant (*I*^*2*^ < 50%, *P* > 0.1). The combined DCR was 0.92 (95%*CI*: 0.91–0.94, *P* < 0.05), and RET-TKIs had a significant benefit on the DCR of RET fusion gene-positive NSCLC (Fig. 6).

### Adverse events

Table 3 showed the common adverse effects of RET fusion-positive NSCLC patients treated with RET-TKIs (grade ≥ 3). Among all grade 3 or above adverse events, the most common adverse event was neutropenia, which was reported in 4 studies with a combined incidence of 13% (95%*CI*: 7–21). Otherwise, anaemia also has a combined incidence of 13% (95%*CI*: 5–24) in 4 studies. A total of 5 literature reported any grade 3 or higher adverse events in treated patients, with a combined incidence of 40% (95%*CI*: 30–51). These findings indicated that patients treated with RET kinase selective inhibitors are at risk for grade 3 or higher adverse events, with neutropenia or anemia becoming the most common adverse events. Our study found that other blood component abnormalities occur at a relatively high rate (Table 3, S2-S6 Fig).

**Table 3:** Meta-analysis of adverse events

Adverse events (grade ≥ 3)	Numbers (N)	Amounts (Events/All)	Rates % (95% *CI*)	Heterogeneity *I*^*2*^ (%)	*P* of Rates
Hypertension	7	142/1179	12 (10–14)	21.39	*P* < 0.01^**^
ALT increased	6	74/1129	6 (3–11)	87.61	*P* < 0.01^**^
AST increased	6	58/1129	5 (3–9)	76.51	*P* < 0.01^**^
hyponatremia	5	57/542	6 (1–13)	88.28	*P* < 0.01^**^
lymphocytopenia	5	52/773	6 (5–8)	0.00	*P* < 0.01^**^
fatigue	6	14/1111	1 (0–2)	32.29	*P* < 0.01^**^
QT interval prolonged	5	22/665	3 (2–4)	42.49	*P* < 0.01^**^
Thrombocytopenia	7	47/1179	4 (2–6)	70.55	*P* < 0.01^**^
Leukopenia	5	48/679	7 (5–9)	36.23	*P* < 0.01^**^
Neutropenia	4	109/629	13 (7–21)	80.77	*P* < 0.01^**^
anaemia	4	84/629	13 (5–24)	89.47	*P* < 0.01^**^
Any AEs	5	460/1047	40 (30–51)	90.65	*P* < 0.01^**^

**P < 0.01

## Discussion

NSCLC harboring RET fusions positive would generally predict metastasis risk and poor prognosis caused by a mutation resulting in characteristic changes of formed cancer proteins [22] and the effect of fusion partners on RET-encoded cancer proteins [23]. For the treatment of RET fusion-positive NSCLC, chemotherapy is still the most commonly used treatment regimen, and the ORR of platinum-based [15] and pemetrexed [24] chemotherapy studies were 51% and 45%, respectively. However, in a retrospective study of immunosuppressive treatment of RET fusion-positive NSCLC patients, the tumor response rate of 16 patients was only 0% [10]. MKIs could inhibit various tyrosine kinase receptors, such as RET, resulting in a cascade of off-target effects against RET fusion-positive NSCLC [9]. Therefore, to achieve better prognosis for patients, inhibitory drugs Pralsetinib and Selpercatinib with high specificity on RET targets were developed and put into clinical trials. This meta-analysis aimed to evaluate the efficacy and safety of RET-TKIs in treating RET fusion-positive NSCLC.

Firstly, the effect of selective RET kinase inhibitors was discussed from ORR, DCR, and mPFS [25]. Concomitative results showed that RET-TKIs treatment was significantly effective in RET fusion-positive NSCLC patients, with ORR of 67% (95%*CI*: 0.64–0.70) and DCR of 92% (95%*CI*: 0.91–0.94), respectively. Patients treated with RET-TKIs had prolonged mPFS of 16.09 months (95%*CI*: 11.66–20.52). Patients with RET fusion-positive NSCLC treated with RET-TKIs can achieve longer disease-free survival, indicating that RET-TKIs have a good effect on these patients, which may be related to the high specificity of Pralsetinib and Selpercatinib for RET. Selpercatinib has a highly selective inhibitory effect on RET, while its inhibitory effect on other tyrosine kinases is negligible^3^. Selpercatinib has been shown to inhibit KIF5B-RET 60 to 1300 times more than MKIs in engineered cells, and significant tumor regression has been seen in RET fusion-positive tumor mouse models [26]. Typically, RET forms dimer complexes with ligands of the glial cell line-derived neurotrophic factor (GDNF) family leading to phosphorylation of the intracellular tyrosine region, activation of RAS/MAPK, PI3K/AKT, JAK-STAT, and other downstream signals. However, the downstream signals are maladjusted after mutation, resulting in uncontrolled cell amplification and malignant differentiation [27]. Meanwhile, Pralsetinib is 100 times more selective to RET mutation than MKIs, and ten times more capable of inhibiting tyrosine phosphorylation than MKIs [28], Which could makes Pralsetinib theoretically effective against RET fusion-positive tumors. In two multicentre Phase I/II trials conducted by Drilon [29]and Griesinger [19], 69 previously untreated patients with RET fusion-positive NSCLC who received Selpercatinib had an ORR of 84% and mPFS of 22.0 months. Among 247 patients who had received at least platinum-based chemotherapy, ORR was 61%, and mPFS was 24.9 months. Of 233 RET fusion-positive patients receiving Pralsetinib, 64% had an ORR and 16.4 months mPFS, including 72% of 75 previously untreated patients with an mPFS of 13.0 months and 59% of 136 treated patients with an ORR and 16.5 months mPFS. These findings indicated the effectiveness of selective RET inhibitors in treating NSCLC patients with positive RET fusion.

Meta-analysis showed that the HR value of ORR was 0.44 (95%*CI*: 0.35–0.56) in patients who had received other treatments compared with those who had not previously received treatment, indicating that RET-TKIs had a better effect on patients who had not received treatment. However, the mechanism is still needed to further explore. The effectiveness may be related to resistance mutations such as RET gatekeeper mutation [30] or RET solvent front mutations [31] in previously treated patients with MKIs.

To investigate the effectiveness of selective RET inhibitors in patients with RET fusion-positive NSCLC with brain metastases through intracranial ORR. Meta-analysis results showed that RET-TKIs also had a good effect on brain metastases in patients with RET fusion-positive NSCLC, with intracranial ORR reaching 87%(95%*CI*:0.68 ~ 0.99). About 50% of RET fusion-positive NSCLC were associated with brain metastases, and most patients developed brain metastases during conventional therapy, including brain parenchyma [32] and meninges [33]. Pralsetinib [33] and Selpercatinib [34] are both designed to penetrate the blood-brain barrier to exert inhibitory effects, especially Selpercatinib has a good intracranial response, and significant effects can be seen in mouse intracranial tumor implantation models [14]. In the phase, I/II clinical study of RET fusion-positive NSCLC patients conducted by Subbiah [35], patients using Selpercatinib achieved an intracranial ORR of 82%, with an mPFS of 13.7 months. Drilon [29] found that in RET fusion-positive patients treated with brain metastases by Selpercatinib, intracranial ORR reached 85%. Griesinger^27^ reported that ten patients with brain metastases were treated with Pralsetinib, and the intracranial ORR reached 70%. In addition, Zhou [36] found that the ORR in Chinese NSCLC patients with RET mutation who deal with Pralsetinib were 66.7% and 83.3% in previously treated group and untreated group, respectively.

The safety of RET-TKIs was evaluated by adverse events that occurred during treatment in this meta-analysis. Full-grade adverse events associated with drug therapy (TRAEs) were observed in a subset of patients treated with RET-TKIs. In a retrospective analysis of a multi-center study of Selpercatinib treatment with RET fusion-positive NSCLC, Illini [20] found that AEs were present in 43 of the 50 patients enrolled in the study. In a multicenter Phase II trial of Selpercatinib in Chinese patients with RET fusion-positive NSCLC, Lu [37] found AEs in 75 out of 77 patients evaluated for the drug’s safety. In Gainor’s [17] found that 216 of 233 patients treated with Pralsetinib developed AEs, the AEs in most patients were a low-grade adverse reaction that could be controlled and reversed. A combined analysis of adverse events (AEs) (grade 3–4) showed high heterogeneity in outcomes of elevated AST, elevated ALT, hyponatremia, thrombocytopenia, neutropenia, anemia, and overall AEs. Further sensitivity analysis was conducted to explore the source of heterogeneity, and no significant impact on heterogeneity was found. Further analysis of the above data using a randomized model revealed a risk of these adverse events after treatment with RET-TKIs, as shown in Table 3. Griesinger [19] reported that 116 previously untreated patients developed AEs (grade 3–4) with Pralsetinib in 60 patients and 93 out of 165 previously treated patients. In the study of Illini [20], 12 of 50 patients treated with Selpercatinib developed AEs (grades 3–4). The incidence of AEs discontinuation was low, although some patients had decreased doses due to adverse reactions. Lu [37] reported that only three patients’ treatment discontinuation wa related to Selpercatinib.

In comparison, Gainor’s [17] report pointed out that only 14 out of 233 patients receiving Pralsetinib were discontinued due to adverse drug reactions. Hematological AEs have been reported to be an essential cause of drug reduction or even withdrawal in patients, which may be related to the inhibition of hematopoietic stem cell differentiation promoted by RET [38]. For example, Griesinger [19] reported that 46 patients were interrupted due to neutropenia, 27 patients were interrupted due to anemia, 18 and 11 patients were interrupted due to leukopenia and thrombocytopenia, respectively. Lu [39] conducted a retrospective analysis of 38 patients treated with selective RET inhibitors and found that 31.6% developed neutropenia, thrombocytopenia, lymphocytopenia, or anemia. Most of them relapsed after drug reduction, eventually leading to drug withdrawal. Overall, the incidence of grade 3–4 adverse events caused by RET-TKIs is low, and most patients can tolerate drug therapy. Grade 5 AEs observed in most studies, such as pneumonia, multiple organ dysfunction, respiratory failure, and cardiac arrest, were not considered to be associated with drug therapy [16, 17]. Therefore, there are drug-related adverse events in treating RET fusion-positive NSCLC with RET-TKIs, However they are relatively safe overall.

Drilon [29] found that the ORR of Selpercatinib and chemo + immune checkpoint inhibitors were analyzed, and the ORR of Selpercatinib reached 67.6%, while the ORR of chemo + immune checkpoint inhibitors was only 14.7%. Otherwise, a Phase III trial (LIBRETOL-431) compares the effects of Selpercatinib with standard chemotherapy ± pembrolizumab in untreated RET fusion-positive NSCLC [40]. LIBRETOL-431 is the first phase III clinical study launched worldwide on RET-TKIs compared to chemotherapy-immunotherapy in RET-positive patients. The study is ongoing, and we expect it will provide more favorable evidence for Selpercatinib in treating RET fusion-positive NSCLC.

Similarly, different RET fusion types could also affect the effect of RET-TKIs. We focused on untreated patients to exclude the impact of pre-study therapy on fusion genes. Gainor [14] and Griesinger [22] pointed out that Pralsetinib had 79% and 74% ORR in KIF5B-RET positive NSCLC patients and 67% and 85% ORR in CCDC6-RET positive NSCLC patients, respectively. The ORR for other fusion types was 40% and 55%. In addition, Griesinger [22] also counted the ORR of Pralsetinib based on clinical characteristics such as gender and smoking history. Lu [39] analyzed the effects of gender, smoking, and CCDC6 mutation on the overall survival time (OS) of RET-mutated NSCLC patients treated by RET-TKIs in his retrospective study. It was found that the above factors had no statistical significance on OS. Therefore, more detailed research support is needed to understand better the effect of clinical features on improving the prognosis of tumor patients with RET-TKIs.

This meta-analysis still has some limitations. Firstly, in the combined analysis of the results of adverse events, there were many indicators with heterogeneity. Although no articles significantly affecting heterogeneity were found in the sensitivity analysis, it may be related to the small number of included literature and research objects, and most of research types were single-arm studies. Secondly, due to the limited time spent on clinical research of RET-TKIs, the published articles are mostly phase I/II clinical studies, and further experiments are still in progress. Therefore, further high-quality researchare still needed.

## Conclusions

Our results indicate that RET-TKIs have good efficacy and safety for RET fusion-positive NSCLC and intracranial metastases, and previously untreated patients have better drug responses than those who have received treatment. Nevertheless, more research is needed to support our research.

### Electronic supplementary material

Below is the link to the electronic supplementary material.

Supplementary Material 1

Supplementary Material 2

Supplementary Material 3

Supplementary Material 4
