# Supplementary material for: The efficacy and safety of selective RET inhibitors in RET fusion-positive non-small cell lung cancer: a meta-analysis
Source: Invest New Drugs. 2023 Aug 21;41(5):768–76. doi: 10.1007/s10637-023-01390-3 (PMC10560178; doi:10.1007/s10637-023-01390-3)
Supplement: Supplementary file 3 — Supplementary Material 3 [file 10637_2023_1390_MOESM3_ESM.docx]

**S1 Table. NOS of the included literature in the meta-analysis.**

| Study | Selection | Comparability | Exposure | Quality scores |
| --- | --- | --- | --- | --- |
| Lu, 2022 | **** | * | ** | 7 |
| Drilon, 2020 | **** | ** | ** | 8 |
| Gainor, 2021 | **** | ** | ** | 8 |
| Griesinger, 2022 | **** | ** | ** | 8 |
| Drilon, 2022 | **** | ** | ** | 8 |
| Illini, 2021 | **** | ** | * | 7 |
| Meng, 2022 | **** | - | * | 5 |
| Zhou, 2023 | **** | ** | ** | 8 |
