# Supplementary material for: The efficacy and safety of selective RET inhibitors in RET fusion-positive non-small cell lung cancer: a meta-analysis
Source: Invest New Drugs. 2023 Aug 21;41(5):768–76. doi: 10.1007/s10637-023-01390-3 (PMC10560178; doi:10.1007/s10637-023-01390-3)

S1 Fig. A single-limb meta-analysis of ORR in patients with RET fusion-gene positive NSCLC treated by RET-TKIs after different treatments.

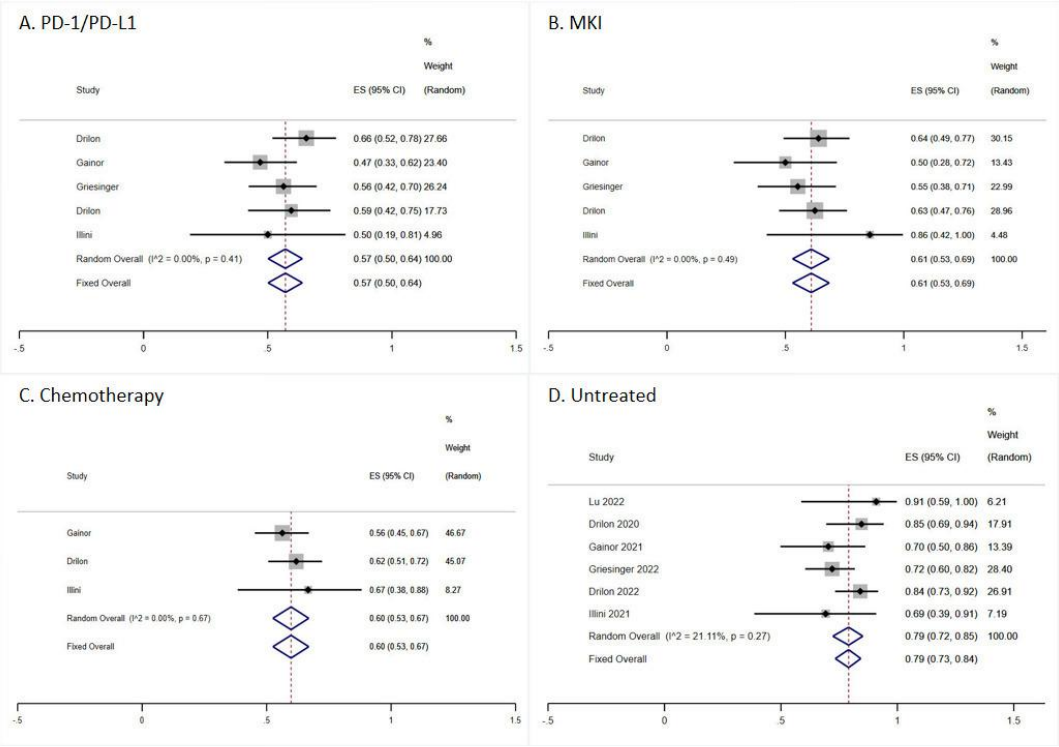

S2 Fig. A single-limb meta-analysis of adverse events in RET-TKIs patients with RET fusion-gene positive NSCLC (hypertension, ALT increased, AST increased, hyponatremia).

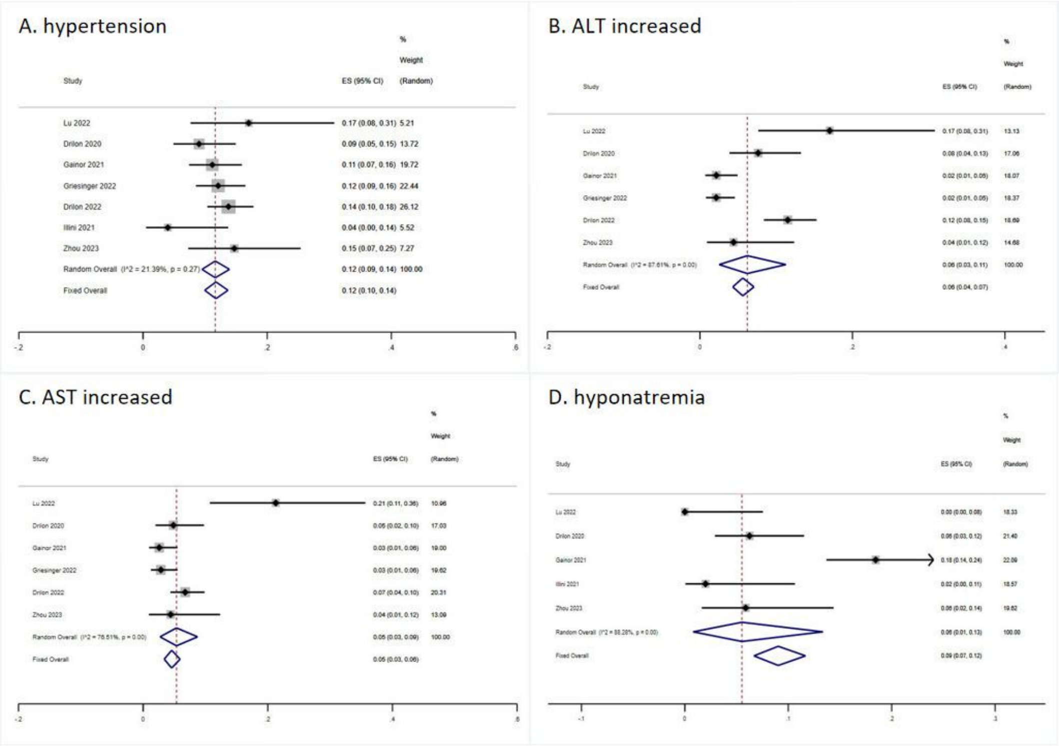

S3 Fig. A single-limb meta-analysis of adverse events in RET-TKIs patients with RET fusion-gene positive NSCLC (lymphocytopenia, fatigue, Thrombocytopenia, Leukopenia).

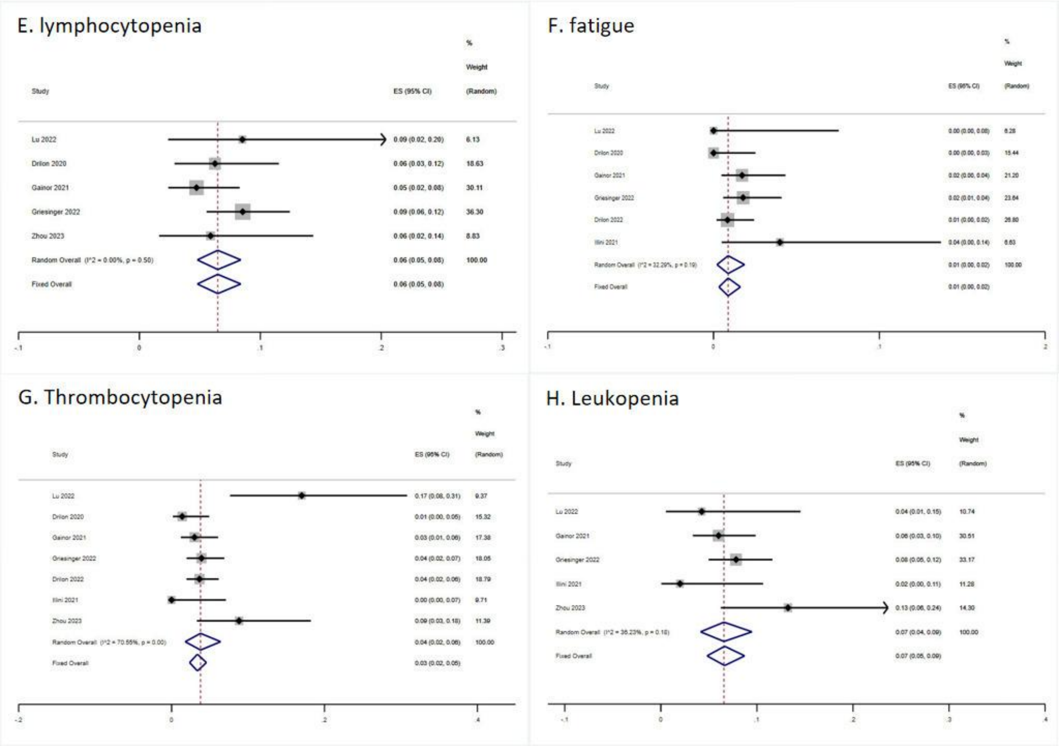

S4 Fig. A single-limb meta-analysis of adverse events in RET-TKIs patients with RET fusion-gene positive NSCLC (Neutropenia, anamia, QT interval prolonged, Any AEs).

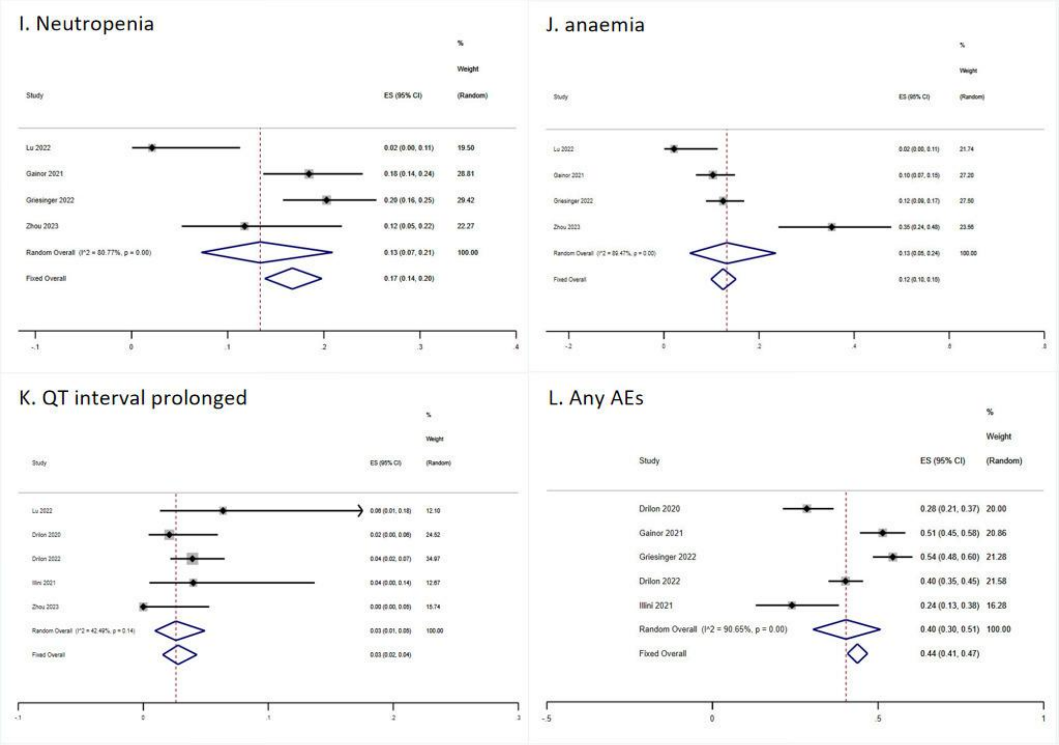

S5 Fig. Sensitivity analysis of adverse events (ALT increased, AST increased, Thrombocytopenia).

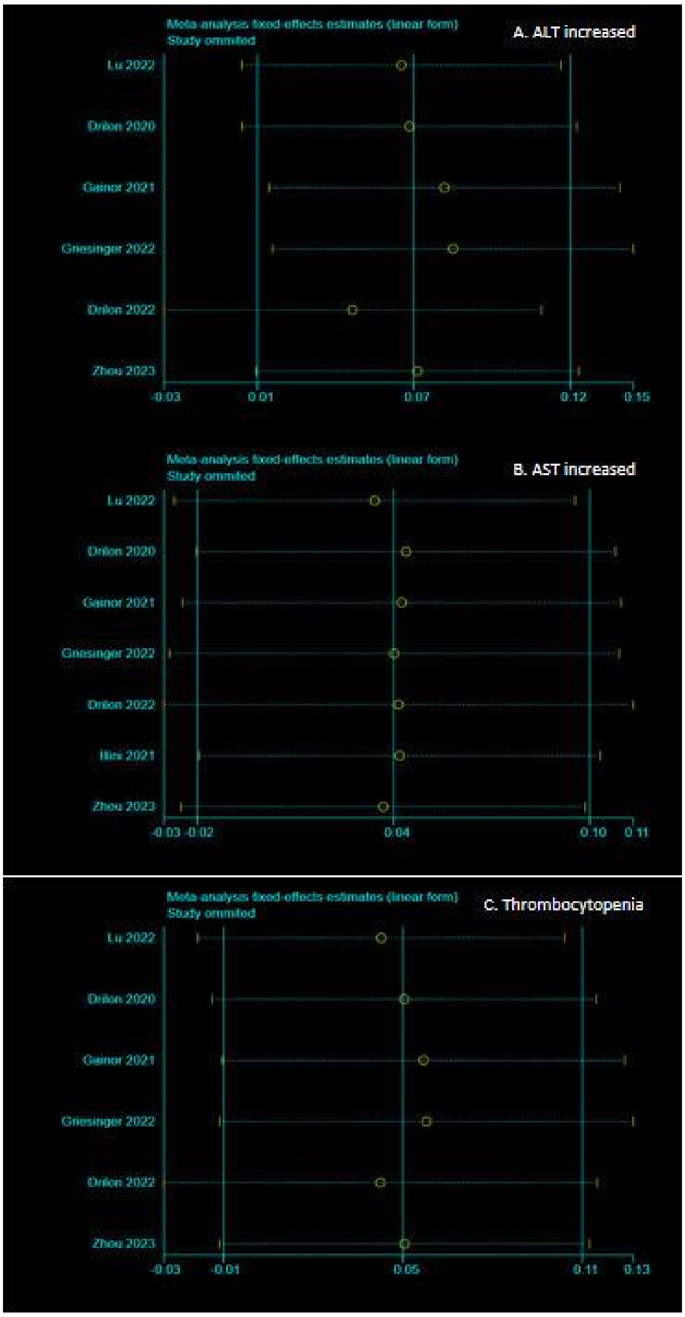

S6 Fig. Sensitivity analysis of adverse events (Neutropenia, anameia, Any AEs).

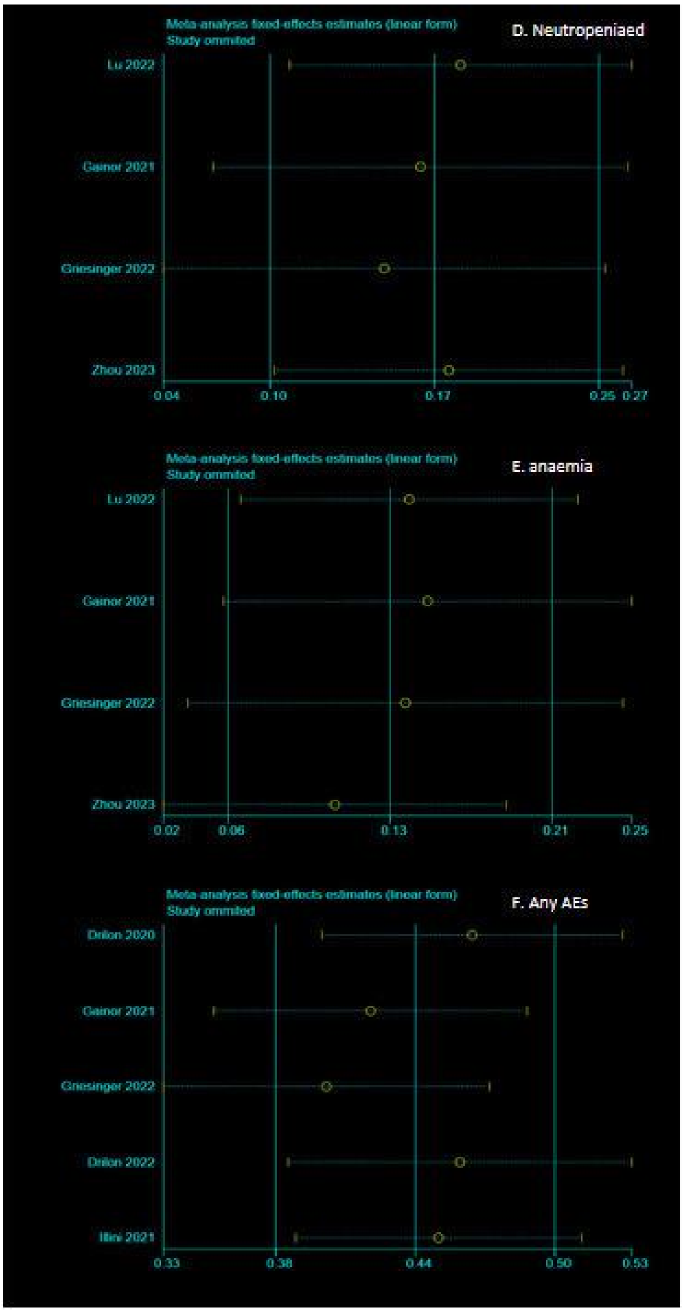

Supplement: Supplementary file 4 — Supplementary Material 4 [file 10637_2023_1390_MOESM4_ESM.pdf]
